# BAF180 regulates cellular senescence and hematopoietic stem cell homeostasis through p21

**DOI:** 10.18632/oncotarget.8102

**Published:** 2016-03-15

**Authors:** Hyemin Lee, Fangyan Dai, Li Zhuang, Zhen-Dong Xiao, Jongchan Kim, Yilei Zhang, Li Ma, M. James You, Zhong Wang, Boyi Gan

**Affiliations:** ^1^ Department of Experimental Radiation Oncology, The University of Texas MD Anderson Cancer Center, Houston, TX, USA; ^2^ Department of Cardiac Surgery, Cardiovascular Research Center, University of Michigan, Ann Arbor, MI, USA; ^3^ Department of Hematopathology, Division of Pathology and Laboratory Medicine, The University of Texas MD Anderson Cancer Center, Houston, TX, USA; ^4^ Department of Molecular and Cellular Oncology, The University of Texas MD Anderson Cancer Center, Houston, TX, USA; ^5^ Program of Genes and Development, The University of Texas Graduate School of Biomedical Sciences, Houston, TX, USA; ^6^ Program of Cancer Biology, The University of Texas Graduate School of Biomedical Sciences, Houston, TX, USA

**Keywords:** BAF180, cellular senescence, hematopoietic stem cell, p21, Gerotarget

## Abstract

BAF180 (also called PBRM1), a subunit of the SWI/SNF complex, plays critical roles in the regulation of chromatin remodeling and gene transcription, and is frequently mutated in several human cancers. However, the role of mammalian BAF180 in tumor suppression and tissue maintenance *in vivo* remains largely unknown. Here, using a conditional somatic knockout approach, we explored the cellular and organismal functions of BAF180 in mouse. *BAF180* deletion in primary mouse embryonic fibroblasts (MEFs) triggers profound cell cycle arrest, premature cellular senescence, without affecting DNA damage response or chromosomal integrity. While somatic deletion of *BAF180* in adult mice does not provoke tumor development, *BAF180* deficient mice exhibit defects in hematopoietic system characterized by progressive reduction of hematopoietic stem cells (HSCs), defective long-term repopulating potential, and hematopoietic lineage developmental aberrations. *BAF180* deletion results in elevated *p21* expression in both MEFs and HSCs. Mechanistically, we showed that BAF180 binds to *p21* promoter, and *BAF180* deletion enhances the binding of modified histones associated with transcriptional activation on *p21* promoter. Deletion of *p21* rescues cell cycle arrest and premature senescence in *BAF180* deficient MEFs, and partially rescues hematopoietic defects in *BAF18*0 deficient mice. Together, our study identifies BAF180 as a critical regulator of cellular senescence and HSC homeostasis, which is at least partially regulated through BAF180-mediated suppression of p21 expression. Our results also suggest that senescence triggered by BAF180 inactivation may serve as a failsafe mechanism to restrain *BAF180* deficiency-associated tumor development, providing a conceptual framework to further understand BAF180 function in tumor biology.

## INTRODUCTION

The SWI/SNF complexes act as evolutionarily conserved chromatin remodeling complexes which utilize energy from ATP hydrolysis to regulate nucleosome sliding, and histone insertion/ejection, resulting in transcriptional activation or repression [1]. In mammals, there are multiple variants of the SWI/SNF complexes, including the BRM-associated proteins (BAF) complex, and the polybromo-containing BAF (PBAF) complex. Each complex consists of ATPases (BRM or BRG1), shared core subunits (SNF5, BAF155, and BAF170), and variant subunits, which are unique to each complex (such that ARID1A or ARID1B is unique to BAF complex, while BAF180, BAF200, and BRD7 are unique to PBAF complex) [2-5]. Notably, various forms of cancers harbor frequent inactivating mutations of several subunits of the SWI/SNF complexes, strongly suggesting that the SWI/SNF complexes function as tumor suppressors in cancer development [6-11]. In addition, the SWI/SNF complex-mediated chromatin remodeling has also been shown to play critical roles in mammalian development, tissue maintenance, and stem cell homeostasis [12-14]. However, it remains less understood how these different subunits of the SWI/SNF complexes contribute to tumor suppression and tissue maintenance in tissue/context-dependent manners.

As mentioned above, BAF180 functions as a variant subunit of the PBAF complex. BAF180 consists of two bromo-adjacent homology (BAH) domains, which mediate protein-protein interactions, and six tandem bromodomains, which recognize mono-acetylated lysine residues on histones [15, 16]. It has been proposed that BAF180 mainly functions to regulate the assembly of the PBAF complex, and/or recruit the PBAF complex to specific loci and regulate the transcription of specific targets [15]. Inactivating mutations of *BAF180* are frequently observed in renal cancer and intrahepatic cholangiocarcinomas [10, 11], suggesting BAF180 is a tumor suppressor in these cancers. However, the exact role and mechanism of BAF180 in tumor suppression remains obscure, and in some cases, controversial. For example, while some studies showed that knockdown of *BAF180* promoted cell proliferation [17, 18], consistent with its proposed tumor suppressor function, another study reported growth suppression phenotype in *BAF180* knockdown cells [19]. Since these studies were conducted in different cell lines with potentially different knockdown efficiencies, it is likely that BAF180 may play a context and cell-lineage specific function in the regulation of cell proliferation. It also highlights the necessity to further clarify its function using a genetically defined complete KO system as used in this study. Finally, the roles of BAF180 in tumor suppression and tissue maintenance *in vivo* remain to be addressed by genetically engineered mouse models.

Cellular senescence, the state of permanent cell cycle arrest, represents an important mechanism in both tumor suppression and tissue maintenance [20-22]. Either oncogene activation or tumor suppressor loss can induce premature senescence, which then serves as a failsafe mechanism to restrict tumor development. On the other hand, it has been shown that senescence promotes stem cell aging, leading to impaired tissue maintenance and repair [22, 23]. Senescence can be regulated by multiple pathways, most notably the p53/p21 and p16/Rb pathways [24, 25]. Senescence is also associated with altered chromatin structures characterized by senescence-associated heterochromatic foci [26]. However, how BAF180-involved chromatin remodeling regulates senescence and senescence-associated tumor suppression and stem cell aging remain largely unknown.

Here, in the course of studying the cellular and organismal functions of BAF180 *via* genetically defined mouse models, we showed that conditional deletion of *BAF180* led to cell cycle arrest and premature cellular senescence in primary MEFs, and decreased stem cell number and function in mouse hematopoietic system. An elevated p21 level was detected in *BAF180* deficient cells. We confirmed that BAF180 directly binds to *p21* promoter and negatively regulates its expression. Deletion of *p21* could rescue cell cycle arrest and premature cellular senescence in *BAF180* deficient MEFs and hematopoietic stem cell (HSC) depletion observed in *BAF180* deficient mouse. Our results suggest that BAF180 regulates transcription of *p21* thereby enabling cells to proliferate and maintaining stem cell homeostasis.

## RESULTS

### *BAF180* deletion triggers premature cellular senescence in MEFs

To study the function of BAF180 in the adult mice as well as in primary mouse embryonic fibroblasts (MEFs), and to circumvent the embryonic lethality phenotype associated with germline nullizygosity [27], we employed a conditional somatic knockout strategy by crossing *BAF180^L/L^* mice [19] with the tamoxifen inducible Cre deleter strain *Rosa26-CreERT2*.

**Figure 1:**
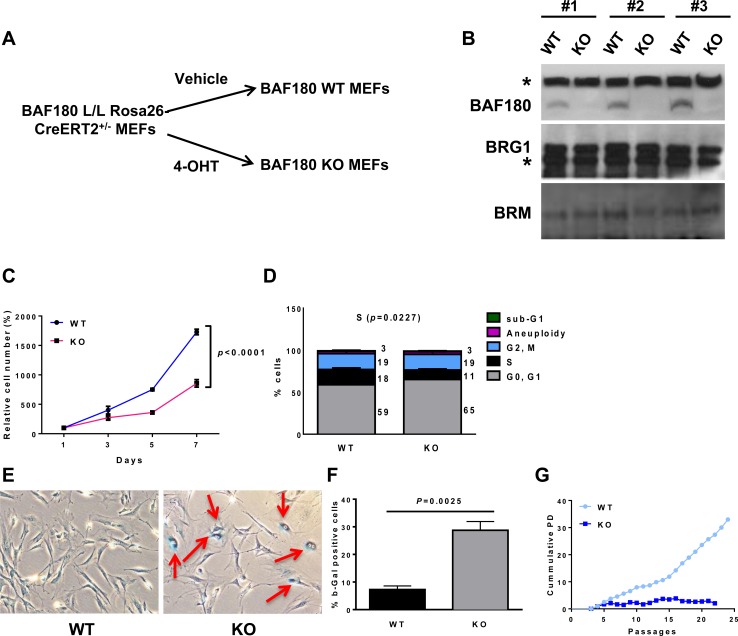
*BAF180* depletion triggers premature cellular senescence in MEFs **A.** A schematic representation of *BAF180* deletion in mouse embryonic fibroblasts. **B.** Immunoblotting of BAF180, BRG1, and BRM in *BAF180* WT and KO MEFs. The asterisk represents a nonspecific band. **C.** Growth curves of *BAF180* WT and KO MEFs. Cells were seeded in triplicate into 12 well plates at a density of 2 × 10^4^ at passage 4 and cell number was determined by a crystal violet staining method (means±SEM, *n* = 3). **D.** Cell cycle status of passage 3 MEFs analyzed by BrdU incorporation and PI staining. Numbers represent the percentage of cells in each cell cycle (Aneuploidy/G2, M/S/G0, G1 from top to bottom; means±SEM, *n* = 4). **E.** Representative images of senescence associated β-galactosidase staining. Cells were plated at passage 4 and cultured for 4 days before staining. **F.** The percentage of β-gal positive cells (means±SEM, *n* = 5). **G.** Cumulative population doublings of *BAF180* WT and KO MEFs starting from passage 4.

Treatment of *BAF180^L/L^*, *Rosa26-CreERT2* MEFs with 4-hydroxytamoxifen (4-OHT) led to near complete loss of BAF180 protein in 4 days (Figure 1A and 1B). Deletion of *BAF180* did not affect the expression level of SWI/SNF ATPase subunits BRG1 or BRM (Figure 1B). Analyses of multiple pairs of matched *BAF180* WT and KO primary MEFs showed that loss of *BAF180* in primary MEFs resulted in delayed cell proliferation and cell cycle arrest in G0/G1 phase with reduced S phase index (Figure 1C and 1D). Compared to *BAF180* WT MEFs at the same passage, *BAF180* KO MEFs exhibited an enlarged and flattened senescence-like morphology with significantly increased senescence associated β-galactosidase activity (Figure 1E and 1F). Correspondingly, *BAF180* KO MEFs exhibited a significantly decreased cumulative population doublings with serial passages. While we could routinely obtain immortalized WT MEFs by 3T3 protocol, it was much more difficult to obtain immortalized *BAF180* KO MEFs (Figure 1G for one representative *BAF180* WT/KO MEF pair).

The cell cycle arrest of *BAF180* deficient cells did not appear to be due to an altered DNA damage response (DDR), since gamma-H2AX and 53BP1 staining did not reveal consistent differences of DNA damage foci among different *BAF180* WT/KO MEF pairs (Figure S1A and S1B). In addition, analysis of metaphase spreads showed that ablation of *BAF180* did not affect chromosomal abnormalities or telomere erosion (Figure S1C) Together, our data reveal that *BAF180* deletion induces premature senescence in primary MEFs, and suggest that it is less likely that DDR and genomic instability represent the prominent underlying mechanism for *BAF180* deletion-induced premature cellular senescence.

### *BAF180* KO mice exhibit defective lymphocytes differentiation, decreased hematopoietic stem/progenitor cells with aging, and impaired repopulation capability

Regulation of cell proliferation and senescence is an important mechanism for maintaining tissue homeostasis [22, 23]. Our aforementioned data thus prompted us to further investigate the impact of *BAF180* loss in adult mice. *BAF180^L/L^*, *Rosa26-CreERT2* and control littermate *BAF180^L/L^* (or *BAF180^+/+^*, *Rosa26-CreERT2*) were injected with tamoxifen for 5 consecutive days at 4 weeks of age, resulting in *BAF180* WT and KO mice. Surprisingly, *BAF180* KO mice were grossly normal. With aging, *BAF180* KO mice exhibited a decrease in the rate of body weight gain compared with WT mice (Figure S2A). *BAF180* KO mice also had defects in lymphocytes development, characterized by reduction in thymus weight and cellularity (Figure S2B and S2C), defective T cell development in thymus (Figure S2D and S2E) and B cell development in bone marrow (Figure S2F and S2G). *BAF180* mutations/deletions have been identified in various human cancers, most notably in renal cell carcinoma [11]. However, *BAF180* KO mice, which have been followed for up to 1 year, did not develop renal cell carcinoma or other type of tumors (data not shown).

**Figure 2:**
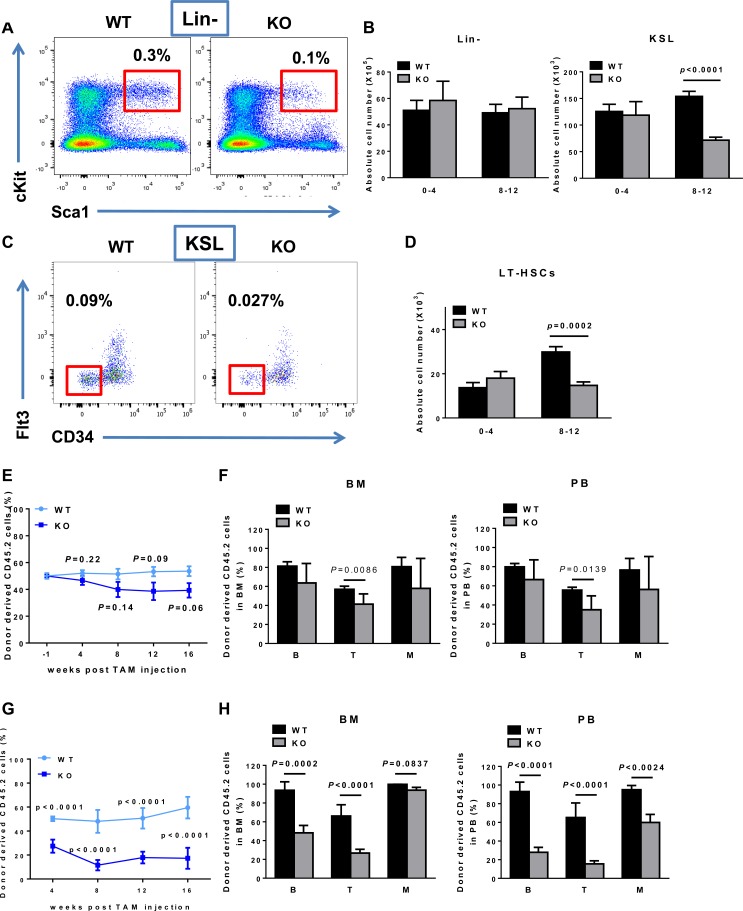
*BAF180* KO mice exhibit decreased absolute number of HSCs and its progenitors with impaired repopulation ability **A.** Flow cytometric analysis of KSL (Lin^−^ Sca-1^+^ c-Kit^+^) cells in bone marrow of *BAF180* WT and KO mice. FACS plots of Lineage negative (Lin-) cells were gated on KSL cells (Red). The numbers indicate the frequency of KSL cells in live bone marrow cells. **B.** Absolute numbers of lineage negative cells (X10^5^, per 1 femur and 1 Tibia) and KSL cells (X10^3^, per 1 femur and 1 Tibia) at 0-4 months and 8-12 months post tamoxifen injection (error bar represents means±SEM, *n* = 2-7; unpaired *t*-test). **C.** Flow cytometric analysis of long-term (LT)-HSCs. KSL cells were gated on CD34^−^ Flt3^−^ population (Red). The numbers indicate the frequency of LT-HSC in live bone marrow cells. **D.** Absolute number of LT-HSCs, CD34^−^ Flt3^−^ KSL. (means±SEM, *n* = 2-7). **E.** Percentage of donor derived cells in peripheral blood of CD45.1 recipients after competitive transplantation (*n* = 8-10). CD45.2 donor cells (either *BAF180* WT or KO) and CD45.1 competitor cells were mixed in a 1:1 ratio and transplanted into lethally irradiated CD45.1 recipients (1×10^6^ cells/recipient). 6 weeks post transplantation (−1 week post tamoxifen injection), blood samples were analyzed and the percentage of donor derived cells were normalized to 50%. **F.** Percentage of donor derived CD45.2 cells were analyzed from total bone marrow and peripheral blood at 16 weeks post transplantation. B (B cells); T (T cells); M (Myeloid cells) (WT: *n* = 6, KO: *n* = 7, 8). **G.** Peripheral blood chimerism of CD45.2 donor derived cells after secondary transplantation (WT *n* = 7, KO *n* = 8). Whole bone marrow cells were isolated from three primary recipients and 3×10^6^ cells were transplanted into a secondary recipient (*n* = 10 recipients per group). **H.** Percentage of donor derived cells in hematopoietic populations 16 weeks after secondary transplantation.

It is well known that appropriate regulation of stem cell function is critical in maintaining tissue and organ homeostasis. We thus examined the roles of BAF180 in the maintenance of HSCs, the best understood adult stem cells [28-30]. Analysis of HSC-enriched KSL cells (Lin^−^ Sca-1^+^ c-Kit^+^) in bone marrow showed no significant difference between young *BAF180* WT and KO mice (0-4 months post tamoxifen injection), yet a significant reduction in both percentage and absolute number of KSL cells was observed after 8 months of post tamoxifen injection (Figure 2A and 2B). Further analysis confirmed the same phenotype in *BAF180* deficient long-term hematopoietic stem cells (LT-HSCs; CD34^−^ Flt3^−^ KSL) (Figure 2C and 2D).

Next we conducted competitive transplantation assay to study the impact of *BAF180* deletion on HSC repopulating ability *in vivo*. Bone marrow cells from CD45.2^+^ *BAF180^L/L^*, *Rosa26-CreERT2* or littermate WT mice were mixed with CD45.1^+^ WT competitor bone marrow cells at 1:1 ratio, and then transplanted into lethally irradiated CD45.1^+^ recipient mice, followed by tamoxifen treatment at 6 weeks post-transplantation to delete *BAF180* in hematopoietic system in the recipient hosts. Analysis of the peripheral blood and bone marrow revealed that *BAF180* KO donor cells were capable of hematopoietic reconstitution (Figure 2E and 2F). Although *BAF180* KO transplants exhibited slightly reduced reconstitution relative to WT controls, the reduction did not reach statistical significance. We then performed secondary transplantation assay by transplanting whole bone marrow cells purified from primary recipients at 16 weeks after tamoxifen injection into secondary recipients. Notably, we observed that *BAF180* KO secondary transplants showed markedly diminished repopulating capability relative to WT controls (Figure 2G and 2H). Together, our results suggest that *BAF180* deletion leads to impaired HSC functionality under aging or stress (serial transplantation) conditions.

### *BAF180* deletion induces p21 in MEFs and HSCs

**Figure 3:**
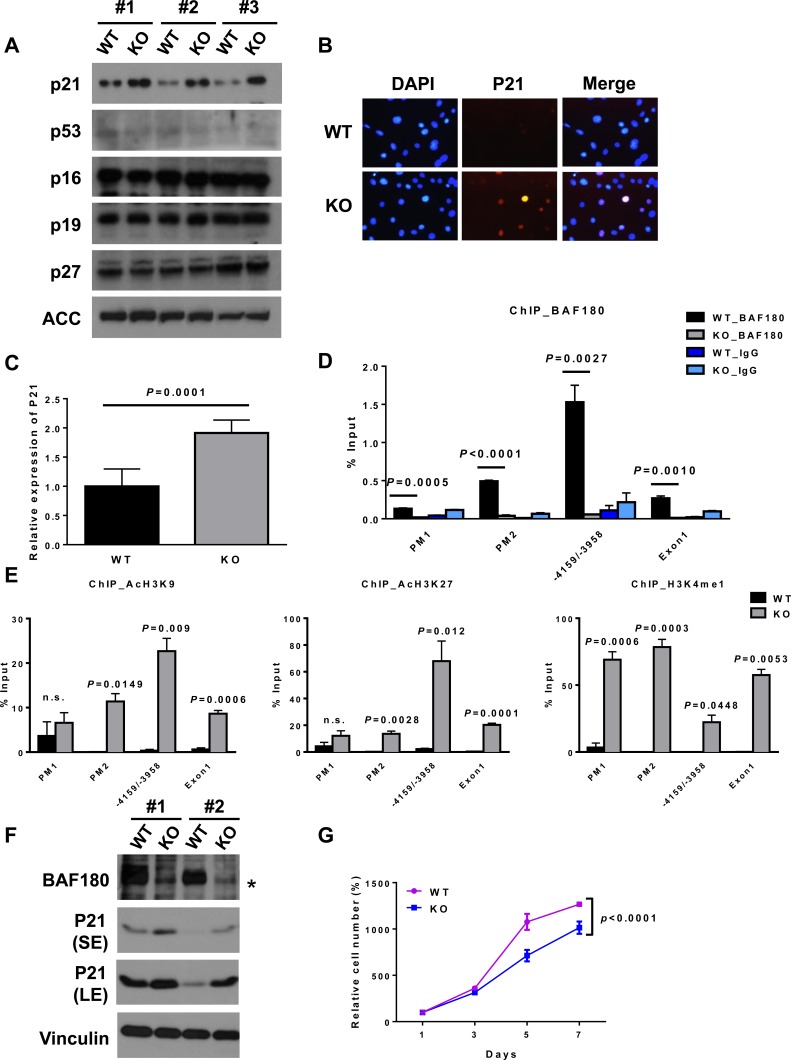
BAF180 negatively regulates p21 expression in MEFs and HSCs **A.** Immunoblotting analysis of cell cycle regulators. Three independent MEFs per genotype were analyzed at passage 6. **B.** Immunofluorescence staining of p21 (Cy3) and DAPI shows elevated p21 level in *BAF180* KO MEFs. **C.** The amount of *p21* transcripts was determined by real-time PCR in purified KSL cell population (means±SD, *n* = 6). **D.** ChIP-qPCR analysis of BAF180 enrichment on *p21* promoter in *BAF180* WT and KO primary MEFs, showing that BAF180 can bind to *p21* promoter at −5681 to −5464 (in the promoter) and −4159 to −3958 nucleotides upstream from the ATG. PM1: promoter −6858/−6610, PM2: promoter −5681 to −5464, Exon 1: +63 to +428 (means±SEM, *n* = 3). **E.** ChIP-qPCR analysis of binding of modified histones, including AcH3K9, AcH3K27 and H3K4me1, on *p21* promoter in *BAF180* WT and KO primary MEFs (means±SEM, *n* = 3). **F.** Immunoblotting analysis of BAF180 and p21 in two pairs of SV40 large T immortalized MEFs. The asterisk represents a nonspecific band. **G.** Growth curves of SV40 large T immortalized *BAF180* WT and KO MEFs (means±SD, *n* = 3).

The aforementioned data prompted us to further study the underlying mechanisms by which BAF180 regulate cellular senescence in MEFs and HSC homeostasis. It is well established that CDK inhibitors, up-stream regulators of p53 and retinoblastoma (Rb) pathways, play critical roles in the regulation of cellular senescence [22]. We thus compared the expression levels of several CDK inhibitors in *BAF180* WT and KO MEFs. Our analysis revealed that the level of p21 was significantly induced in *BAF180* deficient MEFs, whereas the expression levels of its upstream regulator p53 or other CDK inhibitors were not changed (Figure 3A and 3B). Consistent with our results from MEFs, *BAF180* deletion also induced *p21* expression in hematopoietic stem/progenitor cells of 8 month-old mice (Figure 3C).

Chromatin immunoprecipitation (ChIP) assay showed that BAF180 bound to *p21* promoter (Figure 3D). Furthermore, *BAF180* deletion in MEFs led to increased binding of modified histones associated with transcriptional activation, such as H3K9 acetylation, H3K27 acetylation and H3K4 mono-methylation, on *p21* promoter (Figure 3E), suggesting that BAF180 represses *p21* expression through epigenetic regulation of histone modification on *p21* promoter. *BAF180* deletion (by 4-OHT treatment) in SV40 large T antigen-infected (thus p53 defective) *BAF180^L/L^*, *Rosa26-CreERT2* MEFs still resulted in p21 induction and reduced cell proliferation (Figure 3F and 3G), suggesting that BAF180 regulation of p21 and cell proliferation is at least partly p53-independent. Collectively, our results reveal that BAF180 represses *p21* transcription, and *BAF180* deletion results in increased p21 expression in both MEFs and HSCs.

### *p21* deletion rescues cell cycle arrest and premature senescence in *BAF180* deficient MEFs and HSC depletion in *BAF180* deficient mice

**Figure 4:**
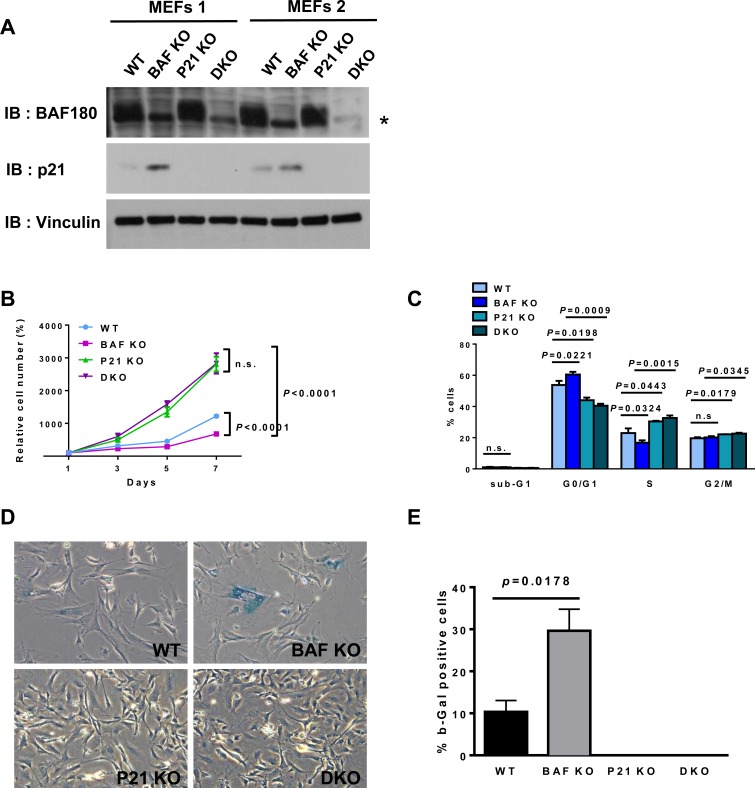
*p21* deletion rescues premature senescence in *BAF180* KO MEFs **A.** Immunoblotting analysis of BAF180 and p21 in passage 6 MEFs. An increased p21 level was observed in *BAF180* KO MEFs. Two independent MEFs lines were used for each genotype. The asterisk represents a nonspecific band. **B.** Growth curves of wild-type, *BAF180* KO, *p21* KO, double KO (DKO) MEFs at passage 4 (*n* = 2, 3). **C.** Cell cycle status of MEFs analyzed by BrdU incorporation and PI staining at passage 3 (means±SD, *n* = 2, 3). **D.** Representative images of senescence associated β-galactosidase staining at passage 4 (*n* = 2, 3). **E.** The percentage of β-gal positive cells (means±SD, *n* = 2, 3). There was no blue stained cell in *p21* KO and DKO MEFs at passage 4.

To determine the functional relevance of *BAF180* deficiency-induced p21 expression to the phenotypic defects observed in *BAF180* deficient MEFs and HSCs, we crossed *BAF180^L/L^ Rosa26-CreERT2* mice with *p21^−/−^* mice, and isolated MEFs from *BAF180^L/L^ Rosa26-CreERT2* and *BAF180^L/L^ p21^−/−^ Rosa26-CreERT2* embryos. MEFs were then treated with either vehicle or 4-OHT to obtain *BAF180^L/L^ p21^+/+^* (WT), *BAF180^−/−^ p21^+/+^* (*BAF180* KO), *BAF180^L/L^ p21^−/−^* (*p21* KO), and *BAF180^−/−^ p21^−/−^* (double KO or DKO) cells. The protein levels of p21 were increased in *BAF180* KO MEFs compared to wild-type MEFs and, as expected, there was no p21 expression in *p21* KO or DKO cells (Figure 4A).

As expected, *BAF180* KO MEFs exhibited decreased, while *p21* KO MEFs showed increased, proliferation rates compared with WT MEFs (Figure 4B). Notably, *p21* deletion fully rescued the proliferation defects observed in *BAF180* KO MEFs, such that the proliferation rate of DKO MEFs was indistinguishable from that of *p21* KO MEFs (Figure 4B), which was further corroborated by the cell cycle profiling analysis (Figure 4C). Full rescue of premature senescence in *BAF180* KO MEFs was also observed in DKO MEFs (Figure 4D and 4E). Together, our results strongly suggest that *BAF180* deletion in MEFs induces p21-dependent checkpoints, resulting in cell cycle arrest and premature senescence.

**Figure 5:**
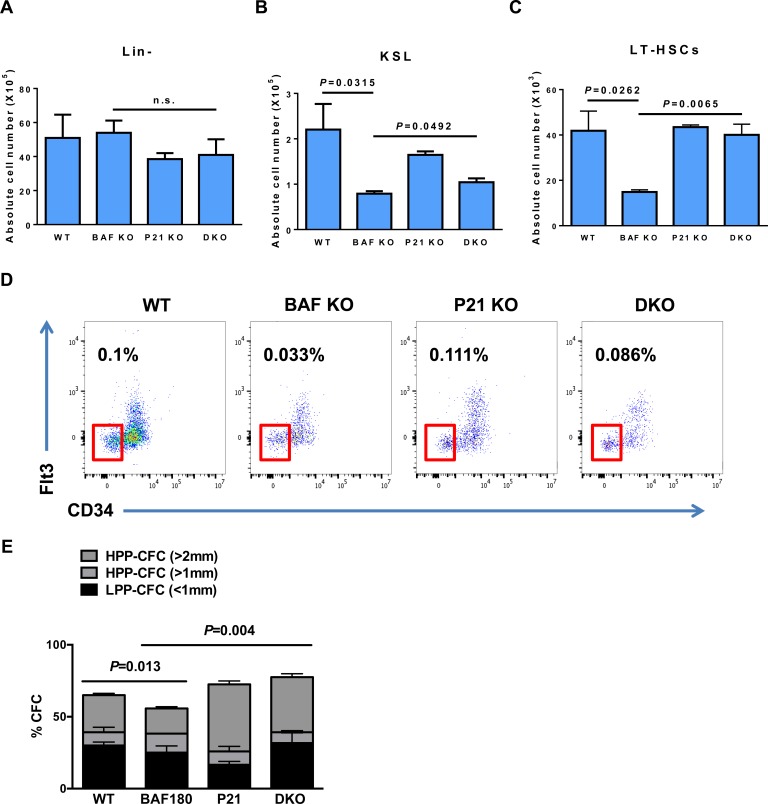
*p21* deletion improves HSC defects in *BAF180* deficient mice **A.** Absolute number of Lineage negative cells (X10^5^) in bone marrow (1 femur and 1 Tibia) of mice of the indicated genotypes. Cells were isolated at 8-10 months post tamoxifen injection (means±SD, *n* = 3, 4). **B.** Absolute number of KSL cells (X10^5^) and **C.** absolute number of LT-HSCs (X10^3^, CD34^−^ Flt3^−^ KSL) per 1 femur and 1 Tibia (means±SD, *n* = 2-4). **D.** Representative FACS profiles of KSL cells of indicated genotypes. FACS plots were gated on LT-HSCs. The numbers indicate the frequency of LT-HSCs in live bone marrow cells. **E.** Single cell CFC assay. Single CD34^−^CD150^+^ KSL cells were sorted into 96 well plates in the presence of mSCF, IL-3 and EPO. Colonies were evaluated at day 14. LPP-CFCs (colony diameter < 1mm), HPP-CFCs (colony diameter > 1mm). 60 single sorted cells per mouse were analyzed (means±SD, *n* = 2, 3).

Since *BAF180* deletion resulted in HSCs depletion and defects in stem cell self-renewal with increase in p21 expression, we examined whether deletion of *p21* in *BAF180* KO mice could rescue HSC defects, as observed in MEFs. We observed that deletion of *p21* did not affect lineage negative cell population (Figure 5A) but improved absolute number of KSL cells in 8-10 month-old *BAF180* KO mice (Figure 5B). *p21* depletion also significantly enhanced LT-HSC population in *BAF180* KO mice in both absolute cell number and frequency (Figure 5C and 5D).

To analyze if loss of *p21* also rescued self-renewal defects in *BAF180* deficient HSCs, we performed *in vitro* colony formation assay from single CD34^−^CD150^+^ KSL cells. BAF180 deficient CD34^−^CD150^+^ KSL cells formed less colonies compared with WT cells, with most notable reduction in high potential proliferating colony forming cells (HPP-CFCs) larger than 2 mm in diameter. Notably, *p21* deletion or *BAF180/p21* double deletion significantly increased the population of HPP-CFCs (> 2mm). In conclusion, our data suggests that *BAF180* deletion results in decreased number and function of hematopoietic stem/progenitor cells, and this defect is at least partly caused by elevated level of p21 in hematopoietic system.

## DISCUSSION

In this study, we show that inactivation of BAF180, a subunit of the SWI/SNF complex, led to premature senescence with G0/G1 cell cycle arrest in MEFs, and resulted in decreased hematopoietic stem/progenitor cells in aged mice with impaired repopulation capability. In addition, we showed that *BAF180* deletion induced p21 expression in both MEFs and HSCs. We provided strong genetic evidence to support the model that *BAF180* deletion-induced p21 is at least partially responsible for the phenotypic defects observed in *BAF180* deficient cells or animals, as deletion of *p21* in the backdrop of *BAF180* deficiency could rescue the premature senescence phenotype in *BAF180* KO MEFs, and significantly rescue the decreased number of HSCs in *BAF180* KO mice. Together, our study suggest a model that, at least in the model system we have studied, BAF180 normally represses p21 expression, and *BAF180* deficiency initiates a p21-dependent checkpoint to induce cell cycle arrest and premature senescence, which likely is responsible for the reduced HSC reserves observed in aged *BAF180* deficient mice.

The aforementioned model is in line with the well-established function of p21 to promote cell cycle arrest and senescence. However, the exact roles of p21 in the regulation of stem cell homeostasis and aging are complex. On one hand, p21 controls HSC quiescence, which is critical in maintaining HSC self-renewal. *p21* deletion resulted in increased HSC cycling and subsequent exhaustion, and defective repopulating potential [31]. On the other hand, it has been shown that activation of p21-dependent checkpoint limits HSC self-renewal in aging telomere dysfunctional mice. Correspondingly, deletion of *p21* improved the repopulating potential and self-renewal of HSCs from mice with dysfunctional telomeres [23]. Similar to the above study conducted in the context of telomere dysfunction, our study suggests that *BAF180* deficiency also likely induces the p21-dependent checkpoint to restrain HSC functionality in aged mice. It is possible that BAF180 is differentially regulated along aging. However, our current data showed that *BAF180* expression does not significantly change during aging in different mouse tissues (Figure S3). The potential mechanism by which BAF180 is regulated under physiological aging process merits further investigation.

In contrast to our data showing that *BAF180* deletion in MEFs led to premature senescence, cell cycle arrest, and increased p21 expression, previous studies showed that knockdown of *BAF180* by shRNA promoted proliferation, or delayed senescence with decreased p21 expression [17, 18]. Since these studies were conducted in different cellular contexts (cancer cell lines in previous studies *vs* primary MEFs and HSCs in this study), it is possible that BAF180 plays context and cell-lineage specific functions in the regulation of cell cycle and p21 expression. For example, certain genetic mutations in cancer cells may turn BAF180 from a p21 repressor to a p21 activator. Alternatively, complete knockout by genetic deletion *vs* partial knockdown by shRNA may result in different outcomes, suggesting the gene dosage of BAF180 may be important for its function.

Recent cancer genomic studies have identified frequent inactivating mutations in *BAF180* gene in various types of human cancers, most notably renal cell carcinoma and intrahepatic cholangiocarcinomas [10, 11]. For example, *BAF180* is mutated in about 40% of clear cell renal cell carcinomas (ccRCCs), a predominant RCC subtype which accounts for up to 75% of all RCC cases, ranking *BAF180* as the second most mutated gene in ccRCC only after *VHL* [11]. Most of these mutations are inactivating mutations. *BAF180* is located on chromosome 3p, a loci frequently deleted in human cancers. These genomic data strongly suggest that *BAF180* is a *bona fide* tumor suppressor in human cancers. However, our current study reveals that broad somatic deletion of *BAF180* in adult mice is not sufficient to drive the development of renal cell carcinoma or other cancers (data not shown). In addition, in contrast to the expected enhanced proliferation or immortalization phenotype from MEFs with deficiency of typical tumor suppressors (such as *p53*), *BAF180* KO MEFs exhibit premature senescence, cell cycle arrest, and are much more difficult to be immortalized (Figure 1). It should be noted that deletion of some tumor suppressors, such as *PTEN* or *VHL*, in primary MEFs also results in premature senescence [32, 33]. It has been proposed that senescence may serve as a failsafe program to restrain tumor suppressor deficiency-induced tumor development, which may explain the lack of tumor phenotypes in our *BAF180* deficient mouse model. Whether combined deletion of *BAF180* and *p21* (or another senescence checkpoint regulator) would promote any tumor phenotype *in vivo* remains to be determined in the future studies. In the case of renal tumor development, since deletion of *VHL* in the mouse is not sufficient to drive renal tumor development [34], it will be interesting to determine whether combined deletion of *BAF180* and *VHL*, the two most frequently mutated tumor suppressors in renal cancer, in the kidney would provoke renal tumor phenotype *in vivo*.

## MATERIALS AND METHODS

### Generation and analysis of mice

The generation of *BAF180^L^* allele was described in the previous publication [19]. *BAF180^L/L^* mice were crossed with tamoxifen-inducible Cre deleter strain, *Rosa26-CreERT2* to generate *BAF180^L/+^ Rosa26-CreERT2^+^* mice. *BAF180^L/+^ Rosa26-CreERT2^+^* mice were further backcrossed six generations with C57BL/6J mice and then F6 progeny were intercrossed to generate mice of the desired genotypes. Tamoxifen treatment of mice to induce Cre recombinase activation and target gene deletion was done as previously described [35-37]. Briefly, tamoxifen (120 ug/gram body weight; Sigma, T5648) was injected into 4 weeks old mice intraperitoneally for five consecutive days. Cre deleter mice (B6.129-Gt(ROSA)26Sortm1(cre/ERT2)Tyj/J), *p21* knockout mice (B6.129S6(Cg)-Cdkn1atm1Led/J) and Ly5.1 recipients for bone marrow transplantation assays (B6.SJL-Ptprca/BoyAiTac) were obtained from The Jackson Laboratory. Tissue samples were fixed in 10% neutral-buffered formalin (Fisher Scientific) overnight, washed with 1XPBS and stored in 70% ethanol at 4°C. Tissues were processed for paraffin embedding and sections were prepared for H&E staining (5 um) and antibody immune staining (3 um). All animal manipulations were performed under MD Anderson IACUC (Institutional Animal Care and Use Committee)-approved protocols.

### Primary and LT-immortalized MEFs

All MEFs were prepared from E13.5 embryos as we previously described [38]. The generation of *BAF180* WT and KO MEFs from *BAF180 L/L, Rosa26-CreERT2* MEFs was conducted as previous described [39, 40]. Briefly, MEFs (passage 0 or 1) were treated with either 200 nM 4-hydroxytamoxifen (4-OHT; Sigma, H7904) or vehicle (ethanol control) for 4 days, resulting in *BAF180* WT and KO MEFs. For immortalized *BAF180* WT and KO MEFs, primary *BAF180 L/L, Rosa26-CreERT2* MEFs were first immortalized by infection of SV40 large T antigen, followed by 4-OHT treatment.

### BrdU incorporation assay

BrdU staining was performed using FITC BrdU Flow kit BD (BD Pharmingen, 559619) according to manufacturer's protocol. Briefly, MEFs were pulsed with 10 uM BrdU for 90 min and trypsinized, fixed and permeabilized. After treatment of DNase for 1 h at 37°C, cells were stained with FITC-labeled anti-BrdU antibody for 20 min at RT, incubated with RNase and PI for 30 min and analyzed with LSRII flow cytometer.

### *In situ* senescence-associated β-gal assay

MEFs (passage 4-6 as indicated) were cultured for 4 days and cellular senescence was determined using a Senescence β-galactosidase staining kit (Cell signaling, #9860). Cells were fixed and β-galactosidase activity was detected at pH6.0. Cells were incubated with X-gal staining solution for 8-12h at 37°C in a dry incubator without CO_2_.

### FACS

Single-cell suspensions were prepared from spleen and thymus by passing cells through a 70 um cell strainer (Falcon). Bone marrow cells from tibias, femurs and spine were crushed with PBS +2% FBS and filtered. After cell counting, cells were stained with following antibody (BD Biosciences, eBiosciences) combinations: Biotin-conjugated lineage marker cocktail (Ter-119, Gr-1, Mac-1, B220, CD4, CD8), HSC (Sca1, c-Kit, CD34, Flt3), T lymphocytes (CD4, CD8, CD25, CD44, Ter119), B lymphocytes (B220, CD4, CD8, CD19, CD43). For KSL cells sorting, cKit positively selected cells were used for staining. Briefly, cells were stained with c-Kit-APC antibody for 30 min on ice, washed with PBS and incubated with anti-APC microbeads for 15 min at room temperature. c-Kit positive cells were collected using a MACS separation column and used for HSC staining. FACS analysis was carried out on LSRII (BD Biosciences) and Gallios (Beckman coulter) at the MDACC Flow Cytometry and Cellular Imaging Core Facility. FACS data was analyzed with Flowjo software V10.0.8.

### Western blot

Cells were lysed in RIPA buffer (50 mM Tris-HCl, pH 7.4, 150 mM NaCl, 0.25 % deoxycholic acid, 1 % NP-40, 1 mM EDTA) containing protease inhibitors (Roche, 4693159001) and phosphatase inhibitors (EMD Millipore, 524625). Protein concentrations were determined by Bradford assay (Bio-Rad, 500-0006) and western blots were performed using 20-40 ug of protein. The following antibodies were used in this study: BAF180 (Bethyl Laboratories, A301-591A, 1:500), p16 (Santa Cruz, sc-1207, 1:1,000), p53 (Santa Cruz, sc-6243, 1:1,000), BRG1 (Santa Cruz, sc-7796, 1:1,000), BRM (Santa Cruz, sc-6450, 1:1,000), p19 (Abcam, Ab80, 1:2,000), p21 (Santa Cruz, sc-6246, 1:1,000), vinculin (Sigma, V4505, 1:50,000).

### Quantitative real-time PCR

RNA from tissue samples was extracted using Trizol (Invitrogen) and RNA from sorted LT-HSC was extracted using PicoPure RNA isolation kit (Life technologies, KIT0204). RNA was treated with RQ1 RNase-free DNase (Promega, M6101) and cDNA was prepared using Superscript III reverse transcriptase (Invitrogen, 18080-051). qRT-PCR was carried out in triplicate for each cDNA sample with TaqMan probe for p21 (CDKN1A, Mm00432448) and following primers : BAF180 (forward 5′- GCCCATATCCTGGAGAAGTTA-3′, reverse 5′-GGCGACCCCTCTTATCAGTA-3′) Ribosomal protein R15 (internal control, forward 5′- CTTCCGCAAGTTCACCTACC-3′, reverse 5′-TACTTGAGGGGGATGAATCG-3′). RT-PCR was run on stratagene Mx3000P (Agilent Technologies).

### Chromatin immunoprecipitation (ChIP) assay

ChIP assays were performed using a commercial kit SimpleChIP^®^ Plus Enzymatic Chromatin IP Kit (Cell signalling) according to manufacture's protocol. Briefly, MEFs were cross-linked at passage 4 and digested chromatin was immunoprecipitated with 2ug of BAF180 antibody (Bethyl Laboratories, A301-591A), 1 ug of H3K9Ac (Abcam, 4441), 1ug of H3K27Ac (Abcam, 4729), 1 ug of H3K4me1 (Abcam, 8895) and 1 ug of IgG (Cell signaling, 2729). The resulting DNA was used for quantitative real-time PCR using primers located on p21 promoter regions. PM1 (F: 5′-GGGCAGTTTTGACATCCTGT-3′, R: 5′-ACAGGCCTCCTCTTCTGTGA-3′), PM2 (F: 5′-AGTGTGGTCCCAGTCAGGTC-3′, R: 5′-AGACGAGGAAAGCAGTTCCA-3′), −4159/−3958 (F: 5′-GCTTCTGGTTGCCAATGTTT-3′, R: 5′-AAACAGGGCACTTGGTTCAC-3′), Exon1 (F: 5′-CGGTCCCGTGGACAGTGAGCAG-3′, R: 5′-GTCAGGCTGGTCTGCCTCCG-3′).

### Competitive transplantation assay

Bone marrow cells from 2 month-old *BAF180* ^+/+^, CreERT2^+/−^ or *BAF180* ^L/L^, CreERT2^+/−^ mice (both CD45.2) were isolated and mixed with bone marrow cells from a CD45.1 mouse at the 1:1 ratio. Cells were then injected into lateral tail vein of lethally irradiated (12 Gy) CD45.1 recipients (1×10^6^ cells/recipient). Six weeks after transplantation, peripheral blood was collected from the recipients to confirm successful reconstitution and recipients were injected with tamoxifen (120 ug/gram body weight) intraperitoneally for five consecutive days. Peripheral blood was collected and analyzed at 4, 8, 12 and 16 weeks post transplantation. For secondary transplantation, total bone marrow cells were isolated from three primary recipients, mixed together in equal proportion and injected into lethally irradiated CD45.1 recipients. Donor chimerism was analyzed from blood every 4 weeks. Three donor mice and eight-ten recipients were used for each transplant.

### HSC single cell colony assay

The assay was conducted as previously reported [41]. Briefly, bone marrow cells were isolated from wild-type, BAF180 KO, p21 KO and BAF180/p21 double KO mice six weeks after tamoxifen injection. Single CD34^−^CD150^+^ KSL cells were sorted into 96-well plates and cultured in serum-free expansion media in the presence of 10 ng ml^−1^ of mouse stem cell factor (mSCF), 10 ng ml^−1^ interleukin-3 (IL-3) and 10 ng ml^−1^ erythropoietin (EPO), 10% fetal bovine serum and penicillin/streptomycin. The number of colony-forming cells was evaluated at day 14.

### Statistics

Statistical analyses were performed using GraphPad Prism 6. The unpaired two-tailed Student's *t*-test was used to calculate *P* values.

## SUPPLEMENTARY MATERIAL FIGURES


