# Supplementary material for: BAF180 regulates cellular senescence and hematopoietic stem cell homeostasis through p21
Source: Oncotarget. 2016 Mar 15;7(15):19134–46. doi: 10.18632/oncotarget.8102 (PMC4991371; doi:10.18632/oncotarget.8102)
Supplement: Supplementary file 1 [file oncotarget-07-19134-s001.pdf]

BAF180 regulates cellular senescence and hematopoietic stem cell homeostasis through p21

Supplementary Material

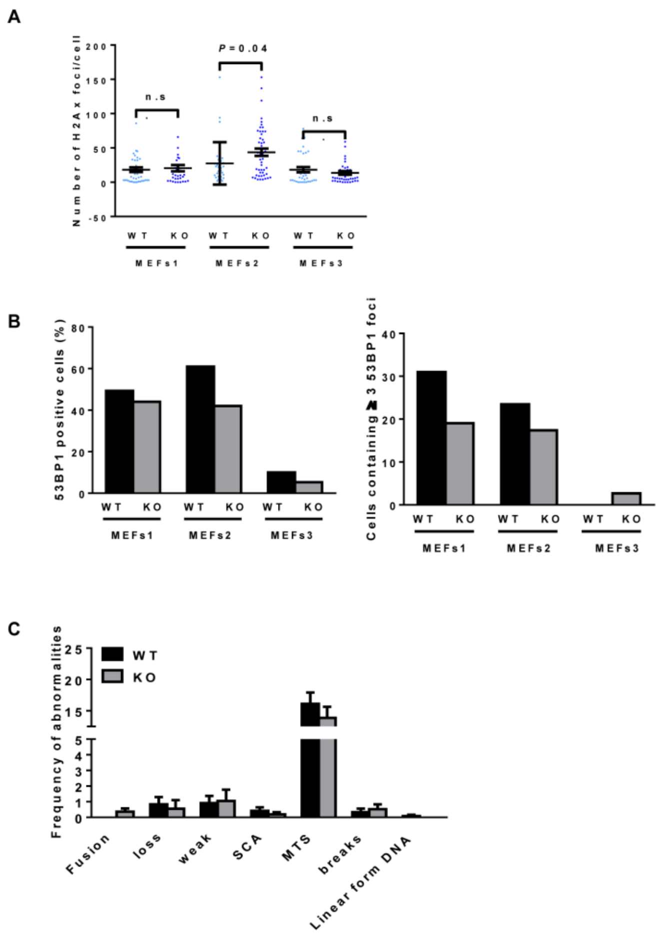

Fig. S1. *BAF180* KO MEFs does not show an altered DNA damage response.

A. The number of gamma H2AX foci was counted from three pairs of matched *BAF180* WT and KO MEFs at passage 5 (means±SD, n=30-50). **B.** Quantification of 53BP1 positive cells (left) and number of cells with more than three 53BP1 foci (right). Three pairs of MEFs at passage 5, 50-100 cells were counted. **C.** Analysis of metaphases obtained from MEFs at passage 5. Telomere PNA FISH was performed on metaphases and chromosomal abnormalities were analyzed from 15-20 metaphases. SCA: sister chromatid association, MTS: multiple telomere signals.

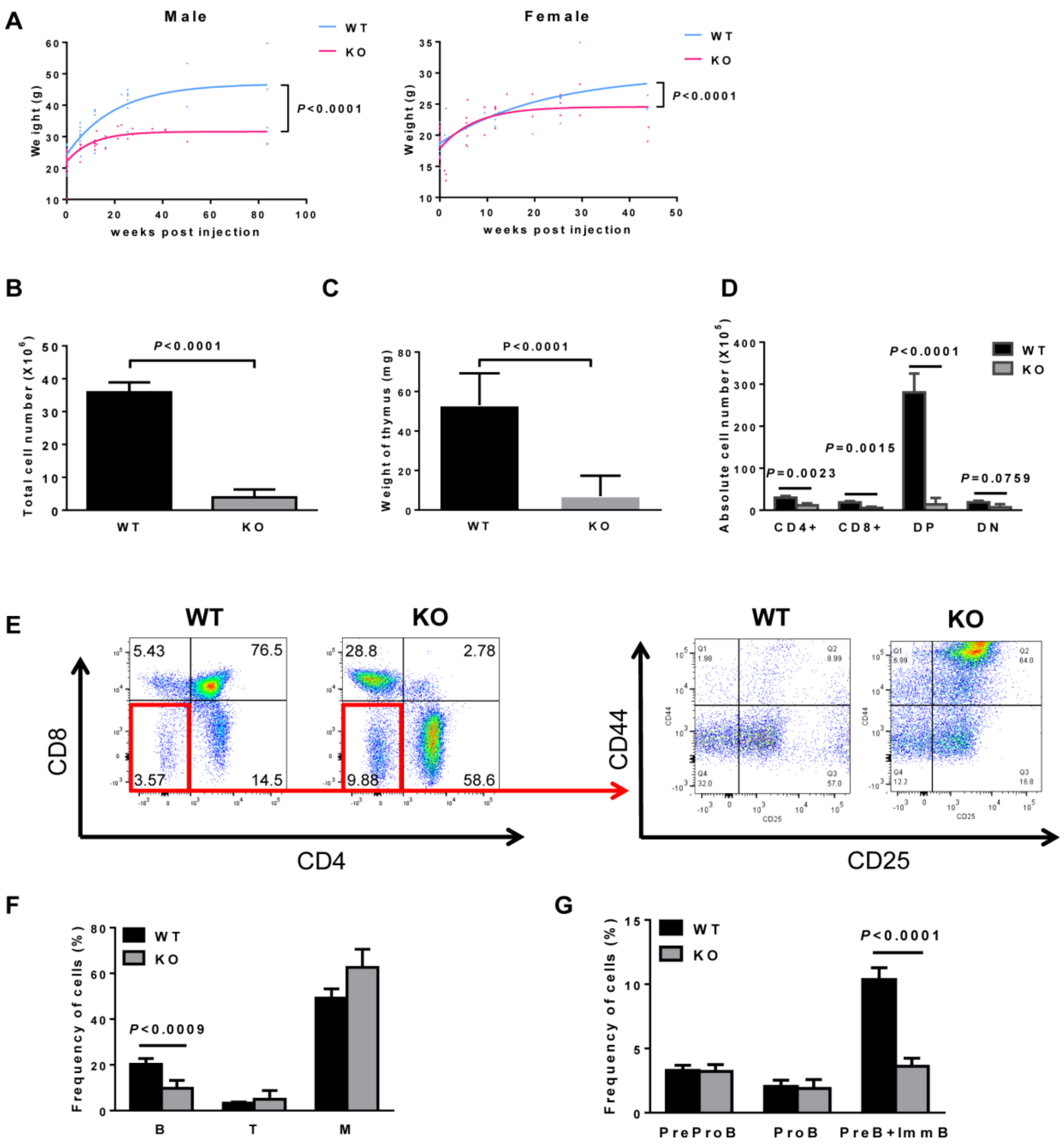

Fig. S2. Ablation of *BAF180* in adult mice results in weight loss and impaired immune cell development.

A. *BAF180* KO mice weighed less than WT mice in both male and female groups. **B.** An acute decrease in thymic weight in *BAF*180 KO mice (WT n=10, KO n=8). **C.** Total number of thymocytes (X 10^6) at 10 days after tamoxifen injection (means±SD, WT n=2, KO n= 7). **D.** Absolute number of $CD4^+$, $CD8^+$, DP ($CD4^+$/$CD8^+$ double positive), DN ($CD4^-$/$CD8^-$ double

negative) thymocytes (X 10^5, means±SD, WT n=2, KO n= 7). **E.** Flow cytometric analysis of thymocytes from *BAF180* WT and KO mice. CD4/CD8 staining (left) and $CD4^-$/$CD8^-$ double negative immature thymocytes were further analyzed by CD25/CD44 (right). *BAF180* KO thymocytes were arrested at $CD25^+$/$CD44^+$ DN2 stage. **F.** B-cells, T-cells and myeloid cells were analyzed in bone marrow of 0-4 month-old of *BAF180* WT and KO mice (means±SEM, WT n=5, KO n=7). **G.** Frequencies of bone marrow B cell subsets were further analyzed by cell surface markers. PrePro B ($B220^+$ $CD43^+$ $CD19^-$ Lin^-), Pro B ($B220^+$ $CD43^+$ $CD19^+$ Lin^-), PreB/ImmB ($B220^+$ $CD43^-$ $CD19^+$ Lin^-), (means±SEM, WT n=5, KO n=7).

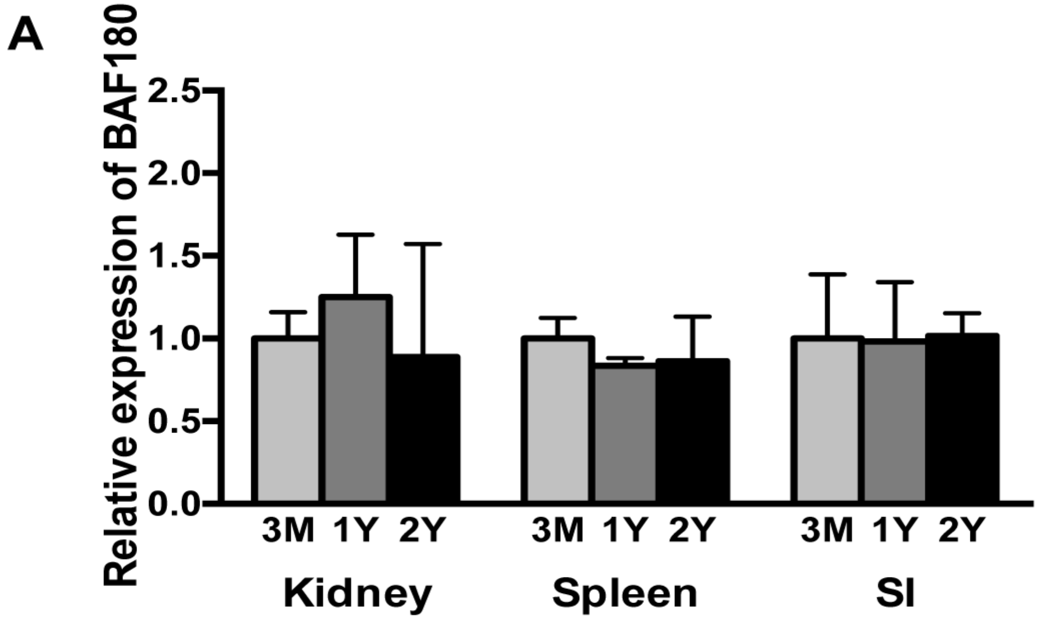

Fig. S3. Expression levels of p21 were not altered under physiological aging process.

A. P21 expression was measured on wild-type mouse tissues by real-time PCR. Tissues was collected at 3 month (3M), 1year (1Y) and 2 year (2Y) old. SI (small intestine). (means±SEM n=2, 5).
